# Effectiveness of physical activity on patients with depression and Parkinson's disease: A systematic review

**DOI:** 10.1371/journal.pone.0181515

**Published:** 2017-07-27

**Authors:** Pei-Ling Wu, Megan Lee, Tzu-Ting Huang

**Affiliations:** 1 Graduate Institute of Clinical Medical Sciences, Nursing, Chang Gung University, Tao-Yuan, Taiwan; 2 Department of biochemistry, University of Washington, Seattle, WA, United States of America; 3 Healthy Aging Research Center School of Nursing, College of Medicine, Chang Gung University, Tao-Yuan, Taiwan; 4 Department of Neurology (Dementia Center), Chang Gung Memorial Hospital Linkou Medical Center, Tao-Yuan, Taiwan; Duke University, UNITED STATES

## Abstract

**Aim:**

In this paper we aimed to systematically review the literature on physical activity’s effect on depressive symptoms in Parkinson disease.

**Background:**

Depression is a common symptom of Parkinson’s disease and is associated with increased disability, rapid progression of motor symptoms, mortality, and adverse effects on Quality of Life.

**Design:**

A systematic review of primary research was undertaken and conducted according to the Preferred Reporting Items for Systematic Reviews.

**Data sources:**

Databases Scopus, Psycho-info, CINAHL, PubMed, and ProQuest Cochrance were searched from January 2006 to June 2017. The language was restricted to English.

**Review methods:**

Abstracts were screened and reviewed against the eligibility criteria (participants’ mean age were ≥ 60 with PD, PA interventions, depression as one of outcome variables, and Randomized Control Trail or quasi-experimental design). Two reviewers appraised the quality of the data extracted. The modified Jadad scale assessed the quality of the methodology of the published papers.

**Results:**

The database search yielded 769 abstracts, 11 of which were included in this review and awarded scores ranging from 3 to 8 (Scale scores range from 0 to 8 points, higher scores indicated better quality) by the raters. These 11 studies included 342 patients and executed 17 kinds of physical activity programs. Results of this review show empirical evidence to support the efficacy of physical activity for the population with Parkinson’s disease. Aerobic training exercise significantly improved the participants’ scores on the Unified Parkinson’s Disease Rating Scale, the Beck Depression Inventory, and the Quality of Life of the patients. Qigong improved scores in UPDRS-III and decreased incidences of multiple non-motor symptoms and depression. Furthermore, a balance-training program, such as Tai Chi, can improve postural stability and Quality of Life.

**Conclusions:**

Physical activity may assuage the degeneration of motor skills and depression as well as increase the Quality of Life of Parkinson’s disease patients, with aerobic training producing the best results. These findings suggest that physical activity, notably aerobic training, could be a good exercise strategy for patients with Parkinson’s disease.

## Introduction

### Depression symptoms in Parkinson’s (PD) disease

PD is a chronic, progressive, and neurodegenerative disease associated with major issues of disability and increased mortality.[1] Non-motor symptoms in PD, such as depression, affects 44%~51.7% of all PD patients.[2, 3] Depression and PD share overlapping symptoms such as reduced facial expression, problems with sleeping, fatigue, psychomotor retardation, and reduced appetite. These similar symptoms may contribute to the underdiagnosis of depression in patients with PD.[4, 5] Moreover, mild depression is an especially common symptom in the early stages of PD and is associated with increased disabilities, rapid progression of motor symptoms, and increased mortality. Furthermore, depression is the main factor affecting Quality of Life (QOL) in PD. Huang et al.[6] suggested that non-pharmacological interventions, such as physical activity, should be the treatment of choice for mild depression.

### Definition of physical activity (PA)

PA is defined as any bodily movement produced by skeletal muscles that result in energy expenditure, which can be measured in kilocalories.[7] PA in daily life can be categorized into occupational, sports, conditioning, household, or other activities.[7–9] The term "exercise" has been used interchangeably with "PA" since the two share many of common elements. Both terms involve bodily movement produced by skeletal muscles that expends energy, are measured by kilocalories ranging continuously from low to high, are positively correlated with physical fitness as the intensity, duration, and increases the frequency of movements.[9, 10] Exercise is PA that is planned, structured, repetitive, and purposeful in the sense that the objective is to improve or to maintain one or more components of physical fitness; examples include swimming, running, and working out at the gym. Tasks regularly performed in the manner described are considered exercise.[7] The PA Guidelines for ACSM recommends that most adults engage in moderate-intensity cardiorespiratory exercise training for ≥30 min, on ≥5 day/week for a total of ≥150 min/wk, vigorous-intensity cardiorespiratory exercise training for ≥20 min/day on ≥3 day/ week (≥75 min/wk1), or a combination of both[10].

### The effects of PA on depression in Parkinson's disease (PD)

PD is among the list of chronic pathological conditions that may benefit from regular PA. In recent years, empirical evidence has shown PA to be an effective strategy to delay or reverse the decline of physical functions and decrease depression in PD affected patients.[11] PA or exercise may have a central effect on depression through an increase in the release of ß-endorphins, in the availability of brain neurotransmitters (such as serotonin, dopamine, and noradrenaline), or in brain-derived neurotropic factors.[12] PA can lead to improvements in self-esteem, self-evaluation, and a sense of achievement.[13] Wipfli et al.[14] found a positive association between PA and self-efficacy. In addition, the increased social interaction experienced during group PA treatments may have positive effects on the patients’ mood[6].

Research evidence shows that PA can reduce depressive symptoms and improve QOL[6, 15] and also improve the self-esteem[15] of older people. Bridle et al.[16] reviewed nine RCTs and discovered that the 3–12 months of PA treatment had an effect on the severity of depressive symptoms in older people overall. However, among PD patients, PA needs to last at least 12 weeks to decrease depressive symptoms.[17] This systematic review adds to the existing body of knowledge by including PA intervention among PD patients studies collected was from an online literature search, and published up to June 2017. Guided by the Preferred Reporting Items for Systematic Reviews and Meta-Analysis Guidelines[18] (S1 Table), the aims of this systematic review were to summarize and critically assess the effect of the physical activity intervention on depressive symptoms amongst PD patients with the evidence collected.

## Materials and methods

### Study eligibility and data extraction

**Fig 1:**
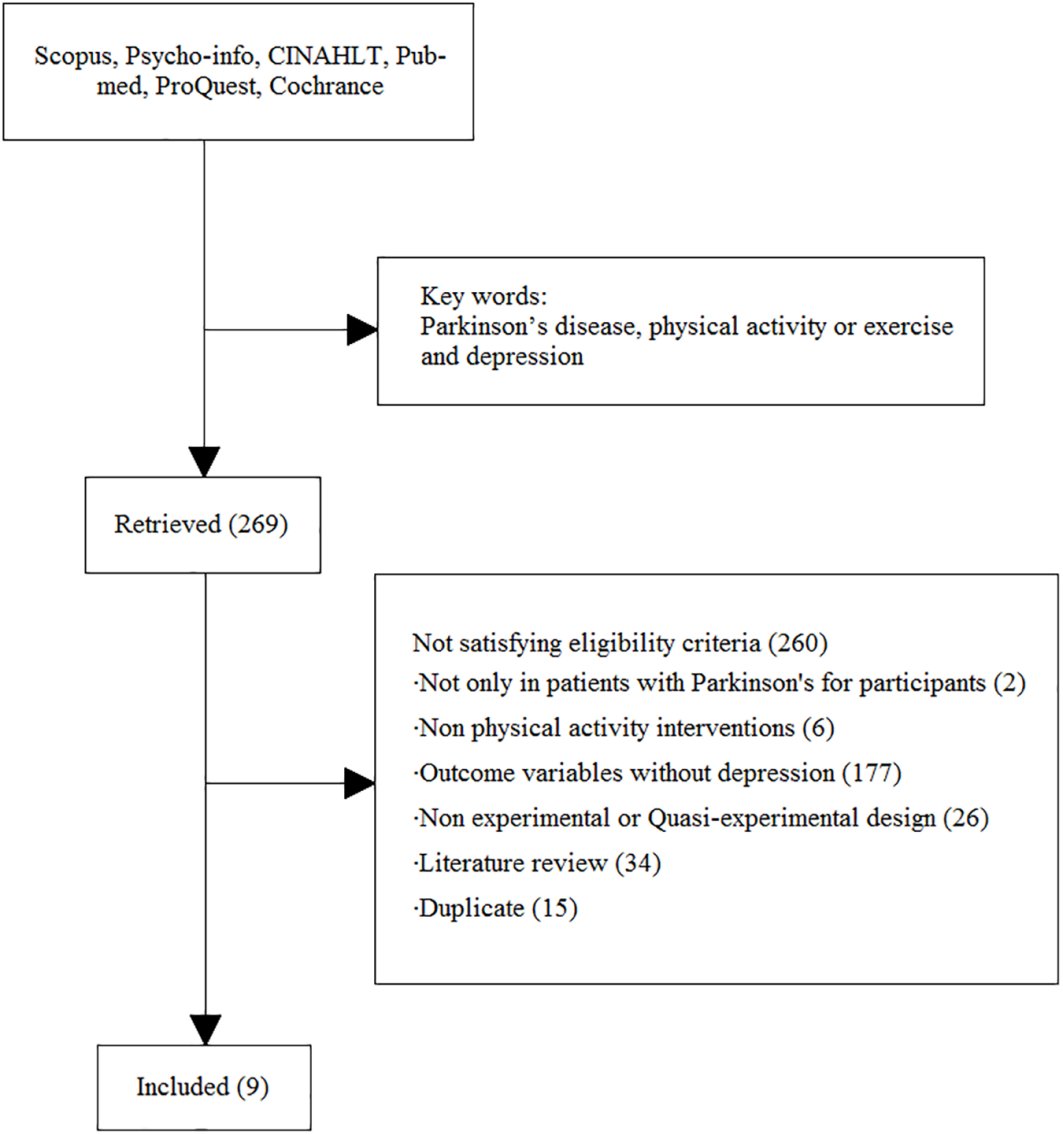
Flowchart of studies.

We searched clinical trial studies regarding PA interventions customized to decrease depressive symptoms in PD patients. A systematic literature search for articles published from January 2006 to June 2017 was performed. Studies searched five electronic databases: Scopus, Psycho-info, CINAHL, Pub Med, and ProQuest Cochrance. The keywords used were “PD”, “PA or exercise”, and “Depression” for a total of 769 articles. The inclusion criteria: (i) participants’ mean age were ≥ 60 with PD; (ii) PA interventions; (iii) Randomized Control Trail or quasi-experimental design. The exclusion criteria is as follows: (i) non-English literature; (ii) no full text; (iii) not only Parkinson’s disease patients participation in the studies; (iv) outcome variables without depression; (v) non-experimental research; (vi) literature review. After the removal of duplicate articles, 11 articles total were included in the final analysis (Fig 1).

### Quality assessment

The selected studies in this review used the modified Jadad scale, the most widely used scale in the world, to assess the studies’ methodological quality.[19] The modified Jadad scale for assessment standards was developed by Greenhalgh[20] and Oremus[21]. The 3-item Jadad scale was developed to measure the quality of clinical trial reports: (i) was the study described as randomized? “yes or no”; award a bonus point if the method of randomization is appropriate (e.g. computer generated, score 2), deduct one point if the method of randomization is inappropriate (score 1)–no randomization score was 0; (ii) was the study described as double-blind? “yes or no”; award a bonus point if the method of double-blinding is appropriate (e.g. identical placebo, score 2), deduct one point if the method of double-blinding is inappropriate (score 1)–no double-blinding score was 0; (iii) Was there a description of withdrawals and dropouts? “yes (score 1) or no (score 0)”. Scale scores can range from 0 to 5 points, with higher scores indicating better quality.[22] Studies with a score of 3 or more were considered high-quality trials and those below 3 were low-quality trials[22].

A modified version of the Jadad scale consists of six items, the three additional questions for the 6-item Jadad scale: (iv) was there a clear description of the inclusion/exclusion criteria? “yes or no” (scored 1 point); (v) was the method used to assess adverse effects described? “yes or no” (scored 1 point); (vi) were the methods of statistical analysis described? “yes or no” (scored 1 point); one point is awarded for each affirmative response. No points are awarded for a negative response. There are six items in the modified version of the Jadad scale, the six items are (i) randomization (“yes” scored 2 points, “no” scored 0); (ii) blinding (“yes” scored 2, “no” scored 0); (iii) description of withdrawals and dropouts (“yes” scored 1 point, “no” scored 0 points); (iv) inclusion/exc1usion criteria (“yes” scored 1 point, “no” scored 0 points); (v) adverse effects (“yes” scored 1, “no” scored 0); (vi) statistical analysis (“yes” scored 1, “no” scored 0).[21] Scale scores range from 0 to 8 points, with higher scores indicating better quality. 1–3 signified low-quality while 4–8 signified high-quality[21].

### Measurement outcome

The outcome variable indicators included physical ability, levels of depression, levels of anxiety, and QOL. The primary outcome was depression.

### Data extraction

Two reviewers extracted data from the chosen papers using structured extraction forms and a third reviewer mediated discussions to resolve any discrepancies. The data analysis included specific details about the populations, interventions, study methods, and outcomes of significance to the review question. When studies are reported to have found significant effects, statistical analyses were run and each study reported required at least a p-value of p<0.05.

## Results

### Classification of study quality

Six among the 11 articles selected earned scores of ≥5 for quality on the modified Jadad scale. Three articles scored 3, two articles scored 4, two articles scored 5, three articles scored 6 and one article scored 1. Two studies were published in 2006, one in 2009, two in 2010, one in 2011, one in 2013, two in 2015, and one in 2016 and 2017. Three studies were randomized (they had described such as envelopes and Computer-generated random allocation table) and three are randomly assigned. There are two double blind studies, two single-blinded, and the other seven studies are non-blinded. Seven of them are I b level of evidence and the other four are II a. The data demonstrates that our systematic review articles are of high quality. Table 1.

**Table 1:** Level of evidence and Jadad quality score.

First author (Year)	Level of evidence	modified Jadad scale						
		Randomization(2)	Blinding (2)	Withdrawals and dropouts (1)	Inclusion/ Exc1usion criteria (1)	adverseeffects (1)	statisticalanalysis (1)	Total
**Burini (2006)**	Ib	2	2	1	1	1	1	8
**Schmitz-Hübsch (2006)**	Ib	2	0	1	1	1	1	5
**Tanaka (2009)**	IIa	0	0	0	1	1	1	3
**Dereli (2010)**	IIa	0	0	1	1	1	1	4
**Smania (2010)**	Ib	0	0	1	1	1	1	6
**Khallaf (2011)**	IIa	0	0	0	1	0	1	3
**Cheon (2013)**	IIa	0	0	1	1	1	1	4
**Dashtipour (2015)**	Ib	2	2	0	1	0	1	6
**Lee (2015)**	Ib	2	0	0	0	0	1	3
**Altmann (2016)**	Ib	2	0	1	1	0	1	5
**Sajatovic (2017)**	Ib	2	1	1	1	0	1	6

### General study characteristics

The database search yielded 769 abstracts, 11 of the 769 are included in this review. These include 5 RCTs and 6 quasi-experimental studies, one of which had no mention of criteria.[23] (Fig 1). A summary of the included studies is detailed in Tables 2 and 3, and 4. The 11 studies include a total of 342 patients. The mean age is 65.02 years old and the ages range from 30~90. There are a total of 138 males and 124 females, among these studies three (N = 71, 9 participants are loss follow up) did not differentiate between genders.[24–26] The sample size in these studies range from 11 to 64, 72.72% (n = 8) studies have ≤ 30 or less subjects. Three studies took place in Europe, three in Asia, three in the United States, one in North Africa, and one in South America. Two studies did not mention the setting in which the intervention was performed[23, 25]; 7 studies took place in a hospital with one home-base exercise in the control group of Dereli’s[27] study, two at exercise facilities and exercise centers.[24, 28] Table 3.

**Table 2:** Summary of general study characteristics.

Study/country	Study population				
	Setting	Sample size	Age	Inclusion criteria	Exclusion criteria
**Burini/Italy**	Neurorehabilitation facility	n = 26	62.7 ~ 65.7	Hoehn & Yahr stage II-III	Severe cognitive impairment, severe neurologic, cardiopulmonary or orthopaedic disorders.
**Schmitz-Hübschy/German**	Outpatient movement disorder clinic	n = 56	63.8	Any stage of disease	1. Recent 1month or planned change medication
					2. Central nervous system disease.
**Tanaka/Brazil**	Laboratory of Physical Activity	n = 20	64.6 ~ 64.8	Hoehn & Yahr stage I–III	Patients in advanced stage of the disease.
**Dereli/Turkey**	Outpatient unit	n = 32	61.3 ~ 66.5	Hoehn & Yahr stage I–III	Change medications used or dosage.
**Smania/Italy**	Rehabilitative gym	n = 64	67.26 ~ 67.64	No other neurological conditions	Mental deterioration and severe dyskinesias.
**Khallaf/Egypt**	Outpatient clinic	n = 30	49 ~ 70	1. Able to walk independently for 6 min.	Neuro musculoskeletal disorder, severe cardiovascular disorders, and cognitive impairment.
				2. Mild-to-moderate disability according to the UPDRS.	
**Cheon/Korea**	Hospital	n = 23	62.3 ~ 65.6	Mild-to-moderate PD.	Severe motor complications, dementia, and psychiatric symptoms.
**Dashtipour/USA**	Outpatient clinic	n = 11	30 ~ 90	1. On a stable dose for the last 28 days.	1. Progression of Parkinsonian features.
				2. No medication changes for the next four months.	2. Severe depression or mental Disorders
**Lee/Korea**		n = 20	68.4 ~ 70.1	1. No cognitive impairment.	
				2. Ambulate independently.	
**Altmann/USA**	Center for Exercise Science	N = 30	62.8 ~ 67.8	1. Diagnosed with idiopathic PD.	1. Participants with secondary or atypical Parkinsonism.
				2. Modified Hoehn and Yahr scale scores ranged between 1 and 3 in the “on” medication state.	
				3. Had a stable response to anti-parkinsonian and/or psychotropic medication.	2. Severe, unpredictable episodes of motor fluctuation.
**Sajatovic/USA**	local exercise facility	N = 30	70	1. Idiopathic PD with Hoehn and Yahr stage III.	1. Mini-Mental State Examination (MMSE) score of 24.
				2. Diagnosis of unipolar major depression with a MADRS score ≥14.	2. Unstable cardiovascular disease.
				3. Stable dose of PD medication for 2 weeks and antidepressant medication (if applicable) for 4 weeks	3. High fall risk, or other uncontrolled chronic conditions.

**Table 3:** Physical activity intervention, duration and intensity.

First author	Research Design			
	Intervention	Duration	Intensity	Instructor
**Burini**	Aerobic training and Qigong	50 mins	3 times per week over 7 weeks	Physical therapist
**Schmitz-Hübsch**	TG: Qigong	Intervention consisted of weekly 60-minute lessons of Qigong	In two courses of 8 weeks with an 8-week pause in-between	An experienced teacher
	GC: no additional intervention			
**Tanaka**	TG: Aerobic exercise (Coordination, Muscular Resistance, and Balance)	60 mins	3times a week, for 6 months	Physical education professionals
	CG: keep their same daily routine			
**Dereli**	Stretching, range of motion, mobility exercises, relaxation exercises, balance, coordination training, gait exercises and breathing exercises	45 mins	10 weeks, three times /week	Physiotherapist
**Smania**	EG: balance training exercise	50 mins	3 days a week for 7 weeks	Therapists
	CG: active joint mobilization, muscle stretching, and motor coordination exercises			
**Khallaf**	G1: aerobic exercise (treadmill) + balance training, weight shifting exercises and muscle strength training	treadmill was increased gradually from 6 to 20 min	At the end of 6 weeks of training	No description
	G2: balance training, weight shifting exercises and muscle strength training			
**Cheon**	G1: The combined stretching-strengthening exercise group	40–50 mins	3 times a week for 8 weeks	Certified instructors
	G2: Tai Chi			
	CG: nonintervention			
**Dashtipour**	G1: General exercise program (combined treadmill plus seated trunk and limb exercises)	60 mins	4 times a week for 4 weeks	Physical therapist
	G2: Lee Silverman Voice Therapy BIG (LSVT BIG therapy)			
**Lee**	EG: virtual reality dance exercise	30 minutes	5 times per week for 6 weeks	
	CG: no exercise			
**Altmann**	G1: aerobic exercise	45 minutes	3 times a week for 16 weeks	certified, fitness specialist
	G2: stretch-balance			
	G3: control (continued their regular daily activities)			
**Sajatovic**	G1: Enhanced EXerCisE thErapy for PD (EXCEED), Group Chronic disease self-management (CDSM), peer support, small group exercise	40 minutes	3 times a week for 12 weeks.	nurse educator certified personal trainer
	G2: self-guided exercise and self-management (SGE), Self-guided CDSM and exercise Assessed			

### Intervention, duration and intensity

#### Types of PA

Within the 11 studies, PA types included: (i) Aerobic Training (AT) and Qigong (Q) (Group1: AT1+QG2, Group2: QG1+AT2) (Burini et al., 2006); (ii) Training Group (TG): Qigong and Control Group (CG): no additional intervention[29]; (iii) TG: Aerobic exercise (multimode exercise program: Coordination, Muscular Resistance, and Balance) and CG daily[30]; (iv) Physiotherapist-supervised vs. Self-supervised groups for an exercise program that includes Stretching, Range of Motion, Mobility, Relaxation, Balance Gait and Coordination Training, and Breathing[27]; (v) Experimental Group (EG): balance training exercise and CG: general physical exercises[31]; (vi) Group (G) 1: aerobic exercise (treadmill) + conventional physiotherapy program, G2: conventional physiotherapy program only[26]; (vii); G1: The stretching-strengthening exercise, G2: Tai Chi and CG: nonintervention[32]; (viii) EG: general exercise and CG: Lee Silverman Voice Therapy BIG (LSVT BIG therapy (Dashtipour et al., 2015); (ix) EG: Virtual reality dance exercise and CG: no exercise[23]; (x) G1:aerobic exercise and G2:stretch-balance (Altmann, 2016). (xi) G1: combined chronic disease self-management (CDSM) with Enhanced EXerCisE thErapy (EXCEED), EXCEED included weekly group CDSM sessions with peer support and guided group exercise, and G2: Self-guided CDSM, exercise and self-management (SGE). A total of 18 different types of PA programs were implemented. Table 3.

#### Duration and intensity

AT for 50 minutes, 3 times per week over a period of 7 weeks for a total of 20 sessions, patients performed Qigong for 20 sessions at the same frequency and intensity as AT[33]; the intervention consisted of weekly 60-minute lessons of Qigong, in two courses of 8 weeks with an 8-week break between the two sessions[29]; aerobic exercise (Coordination, Muscular Resistance, and Balance) for 60 minutes, three times a week, for 6 months[30]; an exercise program (Stretching, Range of motion, Mobility, Relaxation, Balance, Gait and coordination training, Breathing) for 45 minutes, three times a week, for 10 weeks[27]; balance training and general physical exercises (active joint mobilization, muscle stretching, and motor coordination) for 50 minutes, 3 days a week (Monday, Wednesday, Friday) for 7 weeks[31]; increasing an individual’s treadmill walking time gradually from 6 to 20 min, for 6 weeks[26]; stretching and strengthening exercises and Tai Chi, 40–50 minutes, three times a week for 8 weeks[32]; over 4 weeks Dashtipour’s[25], the General Exercise protocol consisted of three parts: a thirty-minute treadmill exercise, a 30-minute seated upper extremity exercise, and the LSVT BIG therapy protocol were encouraged to perform a variety of large amplitude functional movements for 60 minutes with supervision[25]); In Lee’s study, all participants received 30 minutes of neurodevelopment treatment and 15 minutes of functional electrical stimulation 5 times per week for 6 weeks. Additionally, the EG performed 30 minutes of dance[23]; in Altmann’s study, the exercise duration progressed from an initial 20 min per session to the maximum 45 min by increasing exercise time by 5 min each week[24]. In Sajatovic’s study, exercise consisted of 3 times/week sessions, the 1-hour-long group sessions, exercise consisted of fast-paced, low-resistance cycling for 20 minutes followed by strength training for 20 minutes using a progressive sequence of resistance bands. After 12 weeks, individuals continued to exercise on their own. Group 2 was asked to exercise at least 3 times per week and received weekly phone calls during the first 12 weeks to self-report on their exercise.[28] The range of exercise durations is 30–60 minutes, three times a week for 4~12 weeks for PA intervention in these 11 articles. Table 3.

#### Instructor

An instructor did not lead treadmill training and virtual reality dancing. Experienced and certified instructors carried out Qigong and Tai Chi exercises. One study had physical education professionals assist in the aerobic exercise implemented, and the other four studies had physiotherapists aid in the PA treatment. The LSVT BIG therapy was delivered by an LSVT BIG certified physical & fitness therapist, nurse educator and certified personal trainer. Table 3.

### Effect of PA

The following will focus on outcome indicators and variables.

#### Outcome variables

Indicators of outcome variables in the study include UPDRS, symptoms of depression, levels of anxiety, and QOL. Nine of the eleven studies used the UPDRS to measure the physical capabilities of PD patients. All studies measured symptoms of depression, however, their measurement tools varied. Six of the studies used The Beck Depression Inventory (BDI); 2 used the Hospital Anxiety and Depression Scale (HADS) as well as The Hamilton Rating Scale of Depression (HDRS); and two of the studies used the Montgomery-Asperg Depression Rating Scale (MADRS), The Geriatric Depression Scale (GDS-30), and the Beck Anxiety Inventory (BAI). Five studies assessed anxiety symptoms【using HADS, State-Trait Anxiety Inventory, The BAI and Covi Anxiety Scale】. Four studies evaluated QOL as an outcome variable (2 studies used the 39 Item Parkinson's Disease Questionnaire (PDQ-39), one study used the Nottingham health profile and Parkinson's Disease Quality of Life Questionnaire (PDQLQ); and one study used De Bore’s Parkinson's disease quality of life scale (De Bore's PD QOL scale). Table 4.

**Table 4:** Outcome variable.

First author	Outcome variable			
	Depression	Anxiety	Quality of Life	Physical aspect
**Burini**	BDI		PDQ-39	UPDRS-III
**Schmitz-Hübsch**	MADRS		PDQ-39	UPDRS-III
**Tanaka**	HADS	STAI		
**Dereli**	BDI		PDQLQ & NHP	UPDRS
**Smania**	GDS-30			UPDRS
**Khallaf**	HDRS			UPDRS
**Cheon**	BDI		De Bore’s PD QoL scale	UPDRS
**Dashtipour**	BDI	BAI		UPDRS
**Lee**	BDI			
**Altmann**	BDI	BAI		UPDRS Total
**Sajatovic**	MADRS	Covi Anxiety Scale		MDS-UPDRS-III

#### Primary Outcome–depressive symptoms

There are five aerobic exercise programs, two caused no significant changes over time on the BDI[33]; nor on the HADS[30]; another AT (treadmill)[26], Qigong[29], and a combination program Dereli[27], could decrease the symptoms of depression (HDRS, MADRS and BDI, respectively). Balance training, however, did not decrease the score on GDS-30.[31] Cheon[32] found that the combined stretching-strengthening exercise and Tai Chi did not have an effect on BDI.[32] The general exercise program demonstrated positive effects by decreasing BDI[25]. The virtual reality dance had a positive effect on BDI.[23] Lastly, Aerobic exercise and Stretch-balances are a viable intervention for PD that can be protective against worsening depressive symptoms (Altman, 2016; Sajatovic, 2017). Table 4.

#### Secondary outcome–Physical symptoms, anxiety and QOL

The physical aspect aimed to explore motor symptoms and motor disability. Burini et al.[33] found no significant changes on the UPDRS or Brown’s Disability Scale in the AT group; however, a significant increase on the Six-Minute Walking Test (6MWT) and a decrease on the Borg score. In the Qigong group, breathlessness and breathing difficulties decreased significantly. On the contrary, Schmitz-Hübsch et al.[29] found an improvement in UPDRS-III and activities of daily life at 3 months in the Qigong group. Dereli’s exercise program[27] showed a significant improvement on the UPDRS, PA, energy, and functions of daily life. Balance training allowed for dramatic improvements in the Activities-Specific Balance Confidence Scale and the Berg Balance Scale, however, not the UPDRS.[31] The patients in the treadmill and the conventional physiotherapy program showed a significant improvement in walking distance, walking speed, and UPDRS.[26] The combined program of stretching-strengthening and Tai Chi improved physical function and basal cardiovascular endurance more effectively than the intervention with only Tai Chi. Participants in both groups made improvements in their UPDRS score.[32] The effects of the combined treadmill with the seated trunk and limb exercises made improvements on the UPDRS total.[25] The virtual reality dance had a positive effect on balance[23], and aerobic exercise consisted of fast-paced, low-resistance cycling (Sajatovic,2017). Four of the studies reviewed measured anxiety. Tanaka et al.[30] used AT but found no significant changes in levels of anxiety. Dashtipour[25] evinced the effectiveness the general exercise programs had on decreasing anxiety. Aerobic exercise and stretch-balance can decrease anxiety.[24] Burini et al.[33] discovered that AT and Qigong caused no significant changes on QOL (PDQ-39)[29, 33] or on self-efficacy.[33] However, Dereli’s exercise program[27] significantly improved the PDQLQ score, (PDLQ total score, Parkinson’s symptoms, and emotional function). The combined stretching-strengthening exercise performed better in the social domain of QOL, and the Tai Chi fared better in the emotional domain (De Bore’s PD QoL scale).[32] Table 5.

**Table 5:** Outcome.

First author	Main outcome measures	Outcome and Conclusion
**Burini**	UPDRS, B’DS, 6MWT, Borg scale, BDI, PDQ-39	1. Significant increase in 6MWT.
		2. Larger decrease in Borg score.
		3. UPDRS, B’DS, BDI and PDQ-39 scores had no significant changes.
**Schmitz -Hübsch**	UPDRS-III, MADRS, PDQ-39	1. Improved in UPDRS-III and activities of daily living.
		2. Depression scores and the incidence of several nonmotor symptoms decreased in the treatment group.
		3. No significant in QOL.
**Tanaka**	STAI, HADS	1.No depressive symptoms (HADS = 8 or higher)
		2. No significant changes in anxiety.
**Dereli**	UPDRS, BDI, PDQLQ, NHP- total	1. Improved in PDQLQ, NHP-total, BDI and activities of daily living.
**Smania**	UPDRS, GDS	1. No significant improvements in UPDRS.
		2. Significant improvements in the GDS.
**Khallaf**	UPDRS, HDRS	1. More effectiveness in activities of daily living and depressive symptoms.
**Cheon**	UPDRS, BDI, De Bore’s PD QoL scale	1. No improvement in parkinsonian symptoms and depression.
		2. Better in the social domain and the emotional domain of QOL.
**Dashtipour**	UPDRS, BDI, BAI, MFIS	1. Significance improvements in UPDRS total, motor, BDI, and MFIS.
		2. Positive effects of general exercise and LSVT BIG therapy on motor and non-motor symptoms.
**Lee**	BBS, MBI, BDI	1. Positive effect on balance, activities of daily living, and depressive disorder status.
**Altmann**	UPDRS Total, BDI, BAI	1. Aerobic exercise can be protective against increased depressive symptoms, and can improve several non-motor domains. Improved significantly more in the aerobic group than in the stretch-balance group
**Sajatovic**	MADRS	1. Showed significant improvement in MADRS.

BAI (Beck Anxiety Inventory), BDI (Beck Depression Inventory), MFIS (Modified Fatigue Impact Scale), BBS (Berg Balance Scale), MADRS (Montgomery–Asberg Depression, Rating Scale), MBI (Modified Barthel Index).

## Discussion

PD is a chronic, progressive, and neurodegenerative disorder. It is common amongst older adults and increases progressively with age. The prevalence of the disease for those over 60 years is 1%, the numbers increase to 4 to 5% for those 85 years or older.[34] In this study, most of the subjects were over 60 years of age. Four studies are RCT and seven are experimental or quasi-experimental researches. The level of evidence is between Ib-IIa. This study used complete development measurement tools that had high reliability and validity. The MADRS and HDRS have a high degree of validity and internal consistency.[26, 28, 33] Duration and intensity of the PA had an average of 40–60 minutes; the intervals are three times a week, from 4 to 12 weeks.

Depression is the greatest factor affecting the QOL of patients.[35] The effectiveness of PA, such as AT, qigong, general exercise, and balance training on the outcome variable decreases depressive symptoms. Stretching and Tai Chi have no effect on depressive symptoms but can improve physical function and QOL. PA can improve the patients’ clinical status in areas such as PA function, fatigue, depressive symptoms, sleep disorders, and their QOL.[32, 36–40] Our review is consistent with those references; therefore, the PA interventions can be used in clinical practice.

PA interventions can decrease the presence of depressive symptoms in patients with PD, particularly with the implementation of aerobic exercise. This article focuses primarily on recent evidence on the positive effects of exercise on physical function, psychological symptoms, and QOL while highlighting the importance of targeted exercise intervention to maximize the benefits of exercise. Exercise has the potential to lessen both motor (gait, balance, and muscle strength) and non-motor (fatigue, constipation, apathy, depression, and anxiety) symptoms of PD. Effective physical activities such as aerobic exercise (treadmill), exercise programs (stretching, range of motion, movement, relaxation, balance, coordination, gait training exercises, and breathing exercises), physical therapy programs (passive prolonged stretch techniques, balance training, and weight shifting exercises), Qigong, and Tai Chi.[26, 27, 29, 31–33] Aerobic exercise (muscle resistance, coordination, and balance training) can decrease depressive symptoms[24], but has no effect on anxiety[28, 30].

This review article can be utilized when making clinical care decisions. Health benefits of the exercise program are obtained only when 90 minutes of moderate-intensity, weekly exercise is achieved.[41] ACSM guidelines can be applied to patients with PD. The guidelines combined with aerobic exercise, strengthening, and balance training flexion is recommended three times a week for at least 30 minutes.[11] With the requirements met, the PA training program can prevent physical and psychological symptoms. This improvement includes correcting existing pain and loss of energy, preventing recurrence or worsening of symptoms, relieving spasms induced by motor function disability: allowing for normal joint mobility, muscle strength and gait, etc., and re-training when the limb functions can’t be recovered: by teaching and training patients to use its remaining functions. Increased ability to partake in activities in daily life, decrease in depressive symptoms, and better QOL can also be seen if requirements are met.

The comorbidity of anxiety and depression are common clinical symptoms that the PA intervention effectively assuaged. However, in our searching process, only one study included the primary outcome as anxiety. Therefore, we suggested anxiety in patients with PD as the main outcome variable for further research to explore the effectiveness of PA intervention in PD patients with anxiety.

## Conclusion

In this review, we identified that clinicians can be involved in the PA intervention with PD patients, providing evidence for the effectiveness of the Aerobic training (stretching-strengthening exercise, walking, stepping movement, a week 2~3 times, 45–60 minutes every time and lasting for over eight weeks) to help patients minimize physical symptoms and mental symptoms and improve their QOL: all with the main objective of decreasing depressive symptoms.

## Supporting information

S1 Table PRISMA checklist. (PDF)

## References

[1] Rao SS, Hofmann LA, Shakil A. Parkinson’s disease: diagnosis and treatment. Am Fam Physician. 2006;74(12):2046–54. 17186710

[2] Hsu H-Y, Huang T-T, Weng Y-H, Li C-L, Lu C-S. The Inclination to Depressive Mood and Related Factors among Patients with Parkinson's Disease. Journal of Evidence-Based Nursing. 2007;3(3). doi: 10.6225/JEBN.3.3.195

[3] Tagliati M, Chaudhuri K, Pagano G. Prevalence Of Non-Motor Symptoms In Parkinson’s Disease: A Systematic Review With Meta-Analysis (P2. 053). Neurology. 2014;82(10 Supplement):P2. 053.

[4] Dissanayaka NN, Sellbach A, Silburn PA, O'Sullivan JD, Marsh R, Mellick GD. Factors associated with depression in Parkinson's disease. Journal of affective disorders. 2011;132(1–2):82–8. doi: 10.1016/j.jad.2011.01.021 21356559 10.1016/j.jad.2011.01.021

[5] Pachana NA, Egan SJ, Laidlaw K, Dissanayaka N, Byrne GJ, Brockman S, et al Clinical issues in the treatment of anxiety and depression in older adults with Parkinson's disease. Movement Disorders. 2013;28(14):1930–4. doi: 10.1002/mds.25689 24123116 10.1002/mds.25689

[6] Huang T-T, Liu C-B, Tsai Y-H, Chin Y-F, Wong C-H. Physical fitness exercise versus cognitive behavior therapy on reducing the depressive symptoms among community-dwelling elderly adults: A randomized controlled trial. International journal of nursing studies. 2015;2015(52):1542–52. doi: 10.1016/j.ijnurstu.2015.05.013 10.1016/j.ijnurstu.2015.05.013 26105535

[7] Caspersen CJ, Powell KE, Christenson GM. Physical activity, exercise, and physical fitness: definitions and distinctions for health-related research. Public health reports. 1985;100(2):126–31. 3920711 PMC1424733

[8] Global strategy on diet, physical activity and health: physical inactivity: a global public health problem [Internet]. 2015. Available from: http://www.who.int/dietphysicalactivity/factsheet_inactivity/en/.

[9] Global Strategy on Diet, Physical Activity and Health. What is moderate-intensity and vigorous-intensity Physical Activity? [Internet]. 2015. Available from: http://www.who.int/dietphysicalactivity/physical_activity_intensity/en/.

[10] ACSM In The News [Internet]. 2011. Available from: http://www.acsm.org/about-acsm/media-room/acsm-in-the-news/2011/08/01/high-intensity-exercise-best-for-improving-body-composition.

[11] Kolk NM, King LA. Effects of exercise on mobility in people with Parkinson's disease. Movement Disorders. 2013;28(11):1587–96. doi: 10.1002/mds.25658 24132847 10.1002/mds.25658

[12] Dietrich A, McDaniel WF. Endocannabinoids and exercise. British Journal of Sports Medicine. 2004;38(5):536–41. doi: 10.1136/bjsm.2004.011718 15388533 10.1136/bjsm.2004.011718 PMC1724924

[13] Biddle SJ, Asare M. Physical activity and mental health in children and adolescents: a review of reviews. British journal of sports medicine. 2011;45(11):886–95. doi: 10.1136/bjsports-2011-090185 21807669 10.1136/bjsports-2011-090185

[14] Wipfli B, Landers D, Nagoshi C, Ringenbach S. An examination of serotonin and psychological variables in the relationship between exercise and mental health. Scandinavian Journal of Medicine & Science in Sports. 2011;21(3):474–81. doi: 10.1111/j.1600-0838.2009.01049.x 20030777 10.1111/j.1600-0838.2009.01049.x

[15] Park S-H, Han KS, Kang C-B. Effects of exercise programs on depressive symptoms, quality of life, and self-esteem in older people: A systematic review of randomized controlled trials. Applied Nursing Research. 2014;27(4):219–26. doi: 10.1016/j.apnr.2014.01.004 24602398 10.1016/j.apnr.2014.01.004

[16] Bridle C, Spanjers K, Patel S, Atherton NM, Lamb SE. Effect of exercise on depression severity in older people: systematic review and meta-analysis of randomised controlled trials. The British Journal of Psychiatry. 2012;201(3):180–5. doi: 10.1192/bjp.bp.111.095174 22945926 10.1192/bjp.bp.111.095174

[17] Potter R, Ellard D, Rees K, Thorogood M. A systematic review of the effects of physical activity on physical functioning, quality of life and depression in older people with dementia. International journal of geriatric psychiatry. 2011;26(10):1000–11. doi: 10.1002/gps.2641 21905096 10.1002/gps.2641

[18] Moher D, Liberati A, Tetzlaff J, Altman DG, Group P. Preferred reporting items for systematic reviews and meta-analyses: the PRISMA statement. International journal of surgery. 2010;8(5):336–41. doi: 10.1016/j.ijsu.2010.02.007 20171303 10.1016/j.ijsu.2010.02.007

[19] Olivo SA, Macedo LG, Gadotti IC, Fuentes J, Stanton T, Magee DJ. Scales to assess the quality of randomized controlled trials: a systematic review. Physical therapy. 2008;88(2):156–75. doi: 10.2522/ptj.20070147 18073267 10.2522/ptj.20070147

[20] Greenhalgh T. Assessing the methodological quality of published papers. BMJ: British Medical Journal. 1997;315(7103):305–8. 9274555 10.1136/bmj.315.7103.305 PMC2127212

[21] Oremus M, Wolfson C, Perrault A, Demers L, Momoli F, Moride Y. Interrater reliability of the modified Jadad quality scale for systematic reviews of Alzheimer’s disease drug trials. Dementia and geriatric cognitive disorders. 2001;12(3):232–6. 11244218 10.1159/000051263

[22] Jadad AR, Moore RA, Carroll D, Jenkinson C, Reynolds DJM, Gavaghan DJ, et al Assessing the quality of reports of randomized clinical trials: is blinding necessary? Controlled clinical trials. 1996;17(1):1–12. doi: 10.1016/0197-2456(95)00134-4 8721797 10.1016/0197-2456(95)00134-4

[23] Lee N-Y, Lee D-K, Song H-S. Effect of virtual reality dance exercise on the balance, activities of daily living, and depressive disorder status of Parkinson’s disease patients. Journal of physical therapy science. 2015;27(1):145–7. doi: 10.1589/jpts.27.145 25642060 10.1589/jpts.27.145 PMC4305547

[24] Altmann LJ, Stegemöller E, Hazamy AA, Wilson JP, Bowers D, Okun MS, et al Aerobic exercise improves mood, cognition, and language function in parkinson’s disease: results of a controlled study. Journal of the International Neuropsychological Society. 2016;22(9):878–89. doi: 10.1017/S135561771600076X 27655232 10.1017/S135561771600076X

[25] Dashtipour K, Johnson E, Kani C, Kani K, Hadi E, Ghamsary M, et al Effect of exercise on motor and nonmotor symptoms of Parkinson’s disease. Parkinson’s disease. 2015;2015(586378):1–5. doi: 10.1155/2015/586378 25722915 10.1155/2015/586378 PMC4333331

[26] Khallaf M, Fathy H. Effect of treadmill training on activities of daily living and depression in patients with Parkinson's disease. Middle East Current Psychiatry. 2011;18(3):144–8. doi: 10.1097/01.XME.0000398454.71337.40

[27] Dereli EE, Yaliman A. Comparison of the effects of a physiotherapist-supervised exercise programme and a self-supervised exercise programme on quality of life in patients with Parkinson’s disease. Clinical rehabilitation. 2010;24(4):352–62. doi: 10.1177/0269215509358933 20360152 10.1177/0269215509358933

[28] Sajatovic M, Ridgel AL, Walter EM, Tatsuoka CM, Colón-Zimmermann K, Ramsey RK, et al A randomized trial of individual versus group-format exercise and self-management in individuals with Parkinson’s disease and comorbid depression. Patient preference and adherence. 2017;11:965–73. doi: 10.2147/PPA.S135551 28579759 10.2147/PPA.S135551 PMC5449131

[29] Schmitz‐Hübsch T, Pyfer D, Kielwein K, Fimmers R, Klockgether T, Wüllner U. Qigong exercise for the symptoms of Parkinson's disease: a randomized, controlled pilot study. Movement Disorders. 2006;21(4):543–8. doi: 10.1002/mds.20705 16229022 10.1002/mds.20705

[30] Tanaka K, de Quadros AC, Santos RF, Stella F, Gobbi LTB, Gobbi S. Benefits of physical exercise on executive functions in older people with Parkinson’s disease. Brain and cognition. 2009;69(2):435–41. doi: 10.1016/j.bandc.2008.09.008 19006643 10.1016/j.bandc.2008.09.008

[31] Smania N, Corato E, Tinazzi M, Stanzani C, Fiaschi A, Girardi P, et al Effect of balance training on postural instability in patients with idiopathic Parkinson’s disease. Neurorehabilitation and Neural Repair. 2010;24(9):826–34. doi: 10.1177/1545968310376057 21045119 10.1177/1545968310376057

[32] Cheon S-M, Chae B-K, Sung H-R, Lee GC, Kim JW. The efficacy of exercise programs for Parkinson's disease: Tai Chi versus combined exercise. Journal of Clinical Neurology. 2013;9(4):237–43. doi: 10.3988/jcn.2013.9.4.237 24285965 10.3988/jcn.2013.9.4.237 PMC3840134

[33] Burini D, Farabollini B, Iacucci S, Rimatori C, Riccardi G, Capecci M, et al A randomised controlled cross-over trial of aerobic training versus Qigong in advanced Parkinson's disease. Europa medicophysica. 2006;42(3):231–8. 17039221

[34] De Lau L, Giesbergen P, De Rijk M, Hofman A, Koudstaal P, Breteler M. Incidence of parkinsonism and Parkinson disease in a general population The Rotterdam Study. Neurology. 2004;63(7):1240–4. doi: 10.1212/01.WNL.0000140706.52798.BE 15477545 10.1212/01.wnl.0000140706.52798.be

[35] Kadastik-Eerme L, Rosenthal M, Paju T, Muldmaa M, Taba P. Health-related quality of life in Parkinson’s disease: a cross-sectional study focusing on non-motor symptoms. Health and quality of life outcomes. 2015;13(1):83 doi: 10.1186/s12955-015-0281-x 26088201 10.1186/s12955-015-0281-x PMC4474578

[36] Goncalves GB. Using the Nintendo® Wii Fit™ plus platform in the sensorimotor training of freezing of gait in Parkinson’s disease. Arquivos de neuro-psiquiatria. 2013;71(10):828 doi: 10.1590/0004-282X20130135

[37] Lauhoff P, Murphy N, Doherty C, Horgan NF. A controlled clinical trial investigating the effects of cycle ergometry training on exercise tolerance, balance and quality of life in patients with Parkinson’s disease. Disability and rehabilitation. 2013;35(5):382–7. doi: 10.3109/09638288.2012.694962 22747197 10.3109/09638288.2012.694962

[38] Nadeau A, Pourcher E, Corbeil P. Effects of 24 weeks of treadmill training on gait performance in Parkinson disease. Med Sci Sports Exerc. 2014;46(4):645–55. doi: 10.1249/MSS.0000000000000144 24002341 10.1249/MSS.0000000000000144

[39] Pérez CA, Cancela J. Effectiveness of water-based exercise in people living with Parkinson’s disease: a systematic review. European Review of Aging and Physical Activity. 2013;11(2):107–18. doi: 10.1007/s11556-013-0135-7

[40] Volpe D, Signorini M, Marchetto A, Lynch T, Morris ME. A comparison of Irish set dancing and exercises for people with Parkinson’s disease: a phase II feasibility study. BMC geriatrics. 2013;13(1):54 doi: 10.1186/1471-2318-13-54 23731986 10.1186/1471-2318-13-54 PMC3685562

[41] Foulds HJ, Bredin SS, Charlesworth SA, Ivey AC, Warburton DE. Exercise volume and intensity: a dose–response relationship with health benefits. European journal of applied physiology. 2014;114(8):1563–71. doi: 10.1007/s00421-014-2887-9 24770699 10.1007/s00421-014-2887-9

